# Impact of standard enhancement settings of endoscopy systems on performance of endoscopic artificial intelligence systems

**DOI:** 10.1055/a-2530-1845

**Published:** 2025-02-28

**Authors:** Martijn R. Jong, Carolus H. J. Kusters, Querijn N. E. van Bokhorst, Jelmer B. Jukema, Rixta A. H. van Eijck van Heslinga, Kiki N. Fockens, Britt B. S. L. Houwen, Tim J. M. Jaspers, Tim G. W. Boers, Manon van der Vlugt, Evelien Dekker, Fons van der Sommen, Peter H. N. de With, Albert J. de Groof, Jacques J. G. H. M Bergman, on behalf of the BONS-AI Consortium

**Affiliations:** 1 Department of Gastroenterology and Hepatology, Amsterdam Gastroenterology, Endocrinology and Metabolism, Amsterdam UMC, University of Amsterdam, Amsterdam, the Netherlands; 2 Department of Electrical Engineering, Eindhoven University of Technology, Eindhoven, Netherlands

## Abstract

**Background** Artificial intelligence (AI) systems in endoscopy are predominantly developed and tested using high-quality imagery from expert centers. However, their performance may be different when applied in clinical practice, partly due to the diversity in post-processing enhancement settings used in endoscopy units. We evaluated the impact of post-processing enhancement settings on AI performance and tested specific data augmentation strategies to mitigate performance loss.

**Methods** We used a computer-aided detection (CADe) system for Barrett’s neoplasia (6223 images, 906 patients) and a computer-aided diagnosis (CADx) system for colorectal polyps (3288 images, 969 patients), both trained on datasets acquired with Olympus equipment and with limited variability in enhancement settings. The CAD systems were then tested across a wide range of test sets, which comprised the same images, but displayed with different enhancement settings. Both CAD systems were then retrained using image enhancement-based data augmentation. The performance of the adjusted CAD systems was evaluated on the same test sets.

**Results** Both systems displayed substantial performance variability over a range of enhancement settings (CADe: 83 %–92 % sensitivity, 84 %–91 % specificity; CADx: 78 %–85 % sensitivity, 45 %–63 % specificity). After retraining, variability in sensitivity and specificity was reduced to 2 % ( *P* < 0.001) and 1 %, respectively ( *P* = 0.003) for CADe, and 2 % ( *P* = 0.03) and 8 %, respectively ( *P* = 0.19) for CADx.

**Conclusion** The performance of endoscopic AI systems can vary substantially depending on post-processing enhancement settings of the endoscopy unit. Specific data augmentation can mitigate this performance loss.

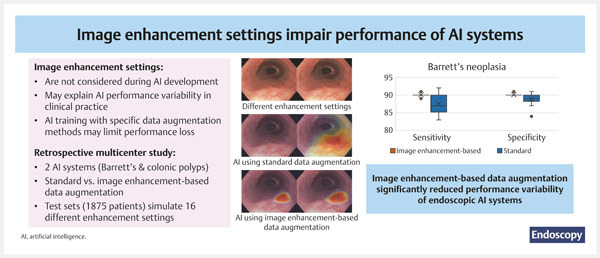

## Introduction

In recent years, advancements in image processing techniques have profoundly impacted the way gastrointestinal endoscopy is performed. High-definition and 4K video technologies now enable unprecedented visualization of vascular and mucosal details. With this enhanced image quality, the bottleneck in disease detection and diagnosis has shifted from visualization to interpretation by the endoscopist. Artificial intelligence (AI) has already shown great promise in assisting this interpretation 1 2 . Nonetheless, the same technological progress that empowers AI also introduces potential challenges that may frustrate successful implementation in daily practice.

**Fig. 1:**
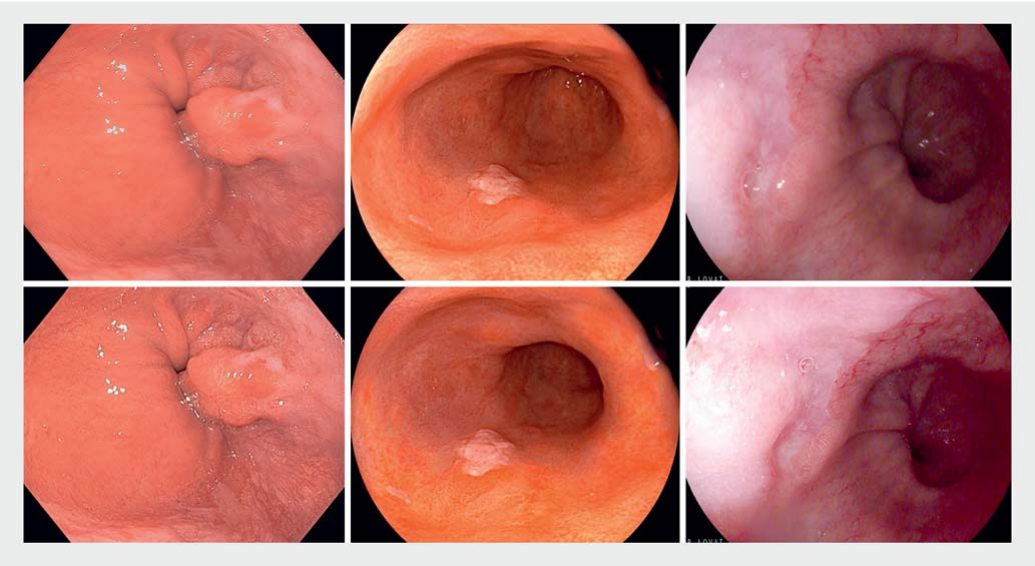
Examples of subtle differences in post-processing settings of endoscopy systems from different manufacturers: enhancement setting type A1 vs. A8 (left; Olympus, Tokyo, Japan), standard settings vs. tone and structure enhancement (middle; Fujifilm, Tokyo, Japan), and white-light endoscopy vs. I-SCAN 1 (right; Pentax, Tokyo, Japan; courtesy of Rehan Haidry).

**Fig. 2:**
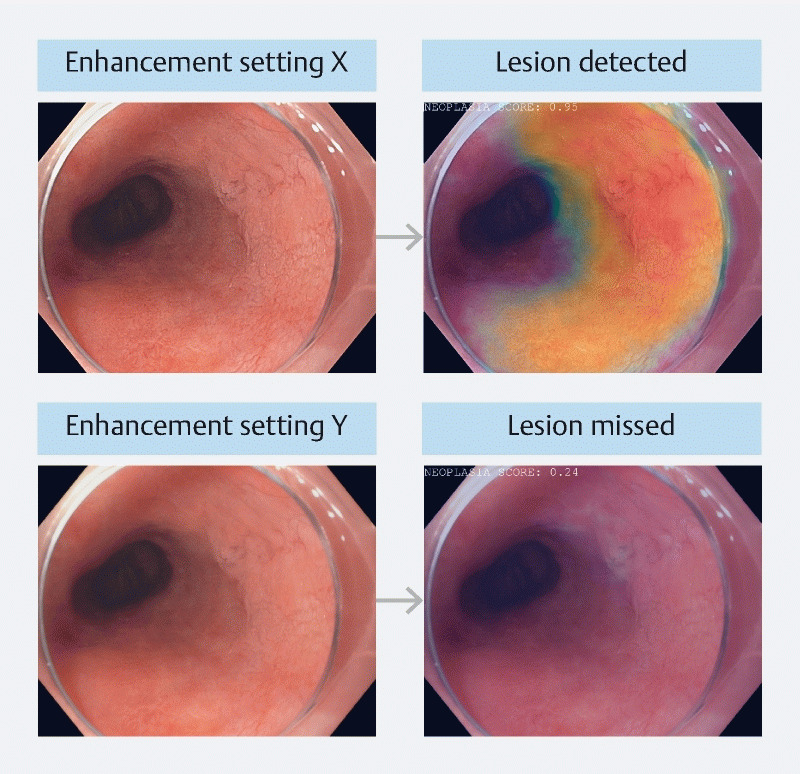
Two separate predictions of a computer-aided detection system for Barrett’s neoplasia. The same case is displayed twice with different levels of image enhancement. While the upper lesion is detected with high confidence, the same lesion projected with a different enhancement setting is missed.

Current endoscopy systems offer a wide range of post-processing enhancement settings that may improve image characteristics such as color, texture, and contrast ( Fig. 1 ). Endoscopists can change these settings according to their own preferences or specific situations. Enhancement settings, while often subtle to the human eye, may significantly impact the accuracy of AI predictions. Fig. 2 exemplifies this issue. This remarkable behavior can be attributed to a phenomenon known as “domain shift.” This term describes the tendency of deep learning systems to perform well on familiar data but deteriorate quickly on data that slightly deviate from the training dataset 3 4 . In the example shown in Fig. 2 , an AI system trained and validated solely on enhancement setting X may perform significantly less well when exposed to enhancement setting Y. In other fields of medicine, studies have shown that domain shift can significantly impact the reliability of AI systems 5 6 7 . The potential risk of domain shift is currently an underexplored issue for AI systems in gastrointestinal endoscopy.

One approach that can help mitigate the effects of domain shift is data augmentation. Data augmentation refers to the technique used to increase the diversity of a training dataset without actually collecting new data. It involves artificially creating new training images by applying transformations to existing images 8 . This can include operations such as rotating, flipping, and cropping images, adjusting contrast, or changing colors. The goal is to make AI systems more robust against new data that differ from the training dataset (i. e. domain shift).

A specialized form of data augmentation is image enhancement-based data augmentation. In this approach, the augmentations focus on transformations that closely align with the changes in post-processing enhancement settings available on endoscopy processors.

In this study, we evaluated the impact of domain shift due to post-processing enhancement settings on performance consistency of two endoscopic AI applications: computer-aided detection (CADe) of Barrett’s neoplasia and computer-aided diagnosis of colorectal polyps (CADx). We trained both CAD systems on datasets with limited variability of enhancement settings using standard data augmentation. We then tested these CAD systems across a wide range of enhancement settings resembling the heterogeneity encountered in daily clinical practice.

Subsequently, we evaluated image enhancement-based data augmentation methods to improve the performance consistency or robustness of both CAD systems against the image heterogeneity of enhancement settings.

## Methods

### Experimental setup

In this study we aimed to evaluate the impact of frequently used image enhancement settings on the performance of endoscopic AI systems. We investigated this based on two prevalent AI applications in endoscopy: CADe for primary detection of early Barrett’s neoplasia using white-light endoscopy, and CADx for optical diagnosis of colonic polyps using narrow-band imaging. For the current study, we redeveloped these two CAD systems based on datasets originating from published studies. First, we retrained these systems using their original datasets, which were acquired with limited variability in enhancement settings. These CAD systems were then tested across a wide range of test sets, each comprising exactly the same images, but displayed with different enhancement settings. Then, both CAD systems were retrained using specific data augmentation methods. The performance of these adjusted CAD systems was then re-evaluated on the same array of test sets.

### Datasets for CAD systems

The CADe dataset consisted of Barrett’s esophagus images collected both retrospectively and prospectively for the development of a previously published CADe system by our own research group 2 9 . All data were acquired in 11 international Barrett’s referral centers. Images were carefully collected and curated according to standardized protocols to adhere to high quality standards. The dataset contained 3339 neoplastic images and 2884 nondysplastic images originating from 637 and 269 Barrett’s patients, respectively. All images were captured with the HQ190 gastroscope and the CV-190 processor (Olympus, Tokyo, Japan) using white-light endoscopy without magnification. The vast majority of images were captured with enhancement setting A5.

The CADx dataset consisted of colonoscopy images collected prospectively for the development of a CADx system for characterization of diminutive colorectal polyps, and is publicly available 10 . Data were acquired in eight Dutch hospitals. All images were collected following a specific workflow to maintain quality consistency across centers. The dataset was separated into a neoplastic group, with adenomas and sessile serrated lesions, and a non-neoplastic group, with hyperplastic polyps. The neoplastic group comprised 2746 images from 736 patients, while the non-neoplastic set contained 542 images originating from 233 patients. Images used in the current study were obtained with 190-series endoscopes and CV-190 processors (Olympus) using narrow-band imaging without magnification. Images were captured with enhancement settings A3 and A5.

**Fig. 3:**
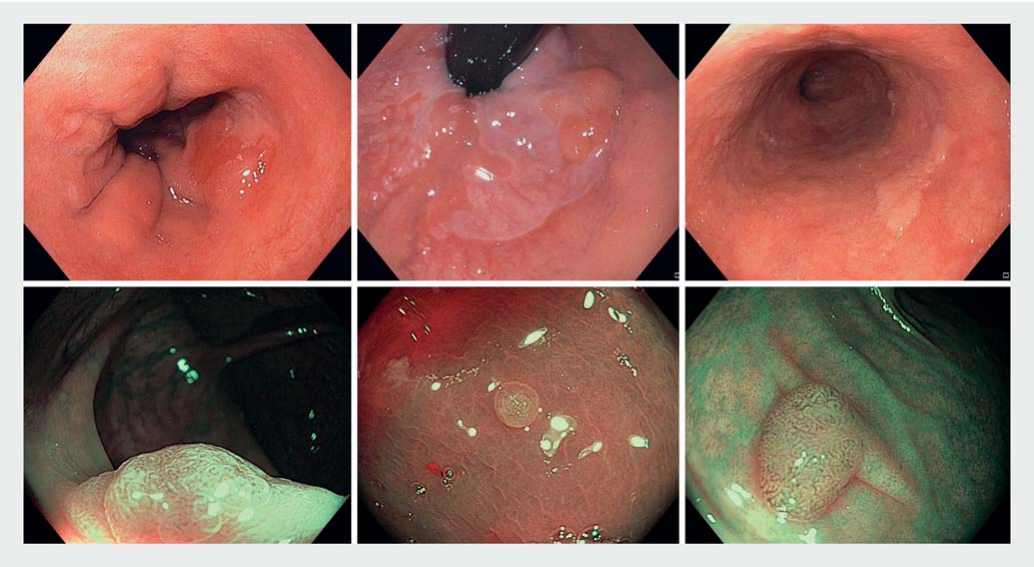
Cases featured in the dataset for computer-aided detection of Barrett’s neoplasia (upper row) and computer-aided diagnosis of diminutive colorectal polyps (lower row).

Examples of both datasets are given in Fig. 3 .

### Enhancement settings

Both datasets were collected using the EXERA III CV190 processor from Olympus. This device offers multiple enhancement settings for both white-light endoscopy and narrow-band imaging. The most frequently used settings are:

- enhancement type A: this setting enhances fine patterns in the image by increasing contrast and sharpness;
- enhancement type B: this setting enhances even finer patterns than type A by more subtly increasing contrast and sharpness.

During an endoscopic procedure, the endoscopist can select one distinct setting and its degree of enhancement (1–8, where a higher number represents stronger enhancement). These settings, as illustrated in **Fig. 1 s** in the online-only Supplementary material, are typically preconfigured by the hospital’s maintenance or technical services team and remain unchanged throughout different procedures.

### Conversion software

In this study, we used a proprietary software tool that was specifically developed to exactly replicate original EXERA III CV190 images with different enhancement settings. By inputting an image and its original enhancement setting, the software can generate equivalent images at other settings. The main differences between these settings are achieved by amplifying or reducing image sharpness and contrast. Comparative examples of original and converted images are provided in **Fig. 2 s** . As shown, the converted images are virtually indistinguishable from original images.

### Data augmentation

In this study, two different data augmentation methods were used.

- Standard data augmentation: this method included geometric transformations (e. g. rotation and flipping), color transformations (e. g. contrast and saturation adjustment), and filtering (e. g. blur and sharpness). These methods are commonly employed to increase the diversity of the training dataset, helping the model to generalize better to new data.
- Image enhancement data augmentation: this method comprised a limited selection of standard augmentations such as geometric transformation in combination with image enhancement-based augmentations. For image enhancement data augmentations, we used the proprietary software tool to augment the training set with images across the complete spectrum of available enhancement settings.

Examples of several data augmentations are given in **Fig. 3 s** .

### Development of CADe and CADx systems

For every dataset (CADe and CADx), two separate CAD systems were developed. Each was trained using either standard or image enhancement-based data augmentation. During training, a patient-based random split was executed, allocating 60 %, 20 %, and 20 % for training, validation, and testing, respectively. A ResNet-50 encoder, initialized with ImageNet pre-trained weights, was employed as this is a widely accepted and commonly used architecture. For all CAD systems, the operating threshold was optimized on its corresponding validation set comprising the same enhancement setting as the training set. Further details are given in the Supplementary material (see Algorithm development).

### Evaluation of CAD systems

To evaluate these CAD systems, we used the following test sets.

- Original test set: this dataset comprised the CAD systems’ original, unaltered test set images, which were acquired using limited variability in enhancement settings.
- Simulated test sets: the software tool was then used to convert the original test set to generate new test sets with different enhancement settings. As the tool offers 16 different enhancement settings (i. e. A1-A8, B1-B8), this resulted in 16 simulated test sets per CAD system.

### Outcome measures

The study outcomes were:

- baseline performance (sensitivity and specificity) of CADe and CADx, trained with standard or image enhancement-based data augmentation, on their original test set;
- performance variability (mean and range of sensitivity and specificity) of CADe and CADx, trained with standard or image enhancement-based data augmentation, across simulated test sets.

### Post hoc analysis

The original training sets of CADe and CADx were acquired using moderate enhancement settings (A3 and A5). In a post hoc analysis, we investigated more extreme enhancement settings. We analyzed scenarios where a CAD system is trained on one end of the spectrum of enhancement settings (e. g. A1) and applied in clinical practice with settings from the opposite end (e. g. A8).

### Statistical analysis

Sensitivity and specificity were chosen as the primary performance metrics in this study. Sensitivity represents the proportion of true positives among all positive cases, while specificity denotes the proportion of true negatives among all negative cases. In the CADe dataset for Barrett’s neoplasia detection, positive cases were defined as images showing neoplastic tissue, while negative cases were nondysplastic Barrett’s images. In the CADx dataset for colorectal polyp characterization, positives included images of neoplastic polyps (adenomas and sessile serrated lesions), and negatives consisted of non-neoplastic polyps (e. g. hyperplastic polyps).

We report sensitivity and specificity for the original test sets to establish baseline performance under the original enhancement settings. Confidence intervals were calculated using the Wilson method. To evaluate variability in performance across different enhancement settings, we tested the systems on an array of simulated test sets generated with varied enhancement settings. For these simulated test sets, we present the median sensitivity and specificity across all settings, alongside the full range of these values to illustrate the extent of performance variability. For a more granular view of this distribution, we provide receiver operating characteristic scatterplots displaying results for each individual simulated test set. To test whether image enhancement-based data augmentation decreased the variability of sensitivity and specificity, we performed a one-sided nonparametric Mood test, using the R package “coin” (R Foundation for Statistical Computing, Vienna, Austria).

## Results

### Performance of CAD systems using standard data augmentation

For Barrett’s neoplasia detection, the CADe system trained with standard data augmentation displayed a baseline performance of 90 % sensitivity and 89 % specificity on its original test set. On the simulated test sets comprising a wide variety of enhancement settings, the CADe system reached median sensitivity and specificity of 87 % and 89 %, respectively. Performance varied substantially between individual sets. Sensitivity ranged between 83 % and 92 % (Δ 9 %) and specificity ranged between 84 % and 91 % (Δ 7 %).

The CADx system for colorectal polyps, trained using standard data augmentation, reached a baseline performance of 79 % sensitivity and 63 % specificity on its original test set. For the simulated test sets, the CADx system displayed median sensitivity and specificity of 79 % and 59 %, respectively. Similarly to CADe, performance variation was clear. Sensitivity ranged from 78 % to 85 % (Δ 7 %) and specificity ranged from 45 % to 63 % (Δ 18 %).

**Fig. 4:**
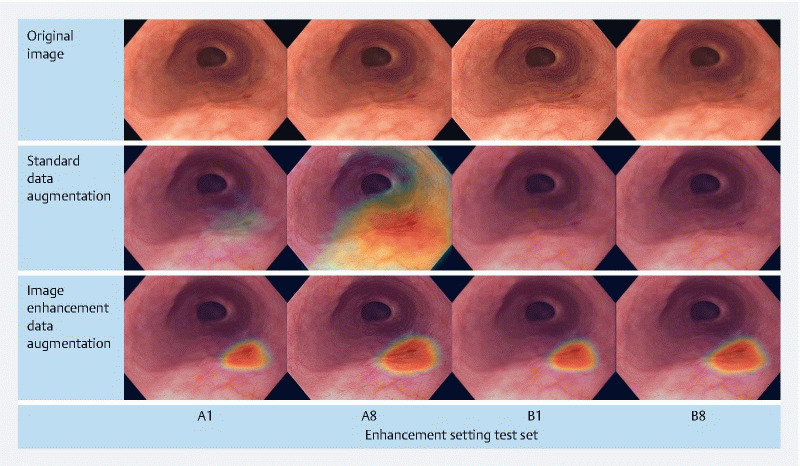
Example case of performance variability of the computer-aided detection system trained with standard data augmentation and image enhancement-based data augmentation. Image enhancement-based data augmentation results in substantially more stable predictions.

An example case of substantial performance variability across different enhancement settings is given in Fig. 4 .

### Performance of CAD systems using image enhancement-based data augmentation

After retraining with image enhancement-based data augmentation, the CADe system showed a baseline performance of 90 % sensitivity and 90 % specificity on the original test set. On the simulated test sets, median sensitivity and specificity were 90 % and 90 %, respectively. Performance variability was significantly lower compared with standard data augmentation. Sensitivity ranged from 89 % to 91 % (Δ 2 %; *P* < 0.001), and specificity ranged from 90 % to 91 % (Δ 1 %; *P* = 0.003).

The retrained CADx system displayed a baseline performance of 78 % sensitivity and 63 % specificity on the original test set. On simulated test sets, the retrained CADx system reached median sensitivity and specificity of 79 % and 60 %, respectively. Performance variability was limited, with sensitivity ranging from 78 % to 80 % (Δ 2 %; *P* = 0.03) and specificity ranging from 55 % to 63 % (Δ 8 %; *P* = 0.190).

**Fig. 5:**
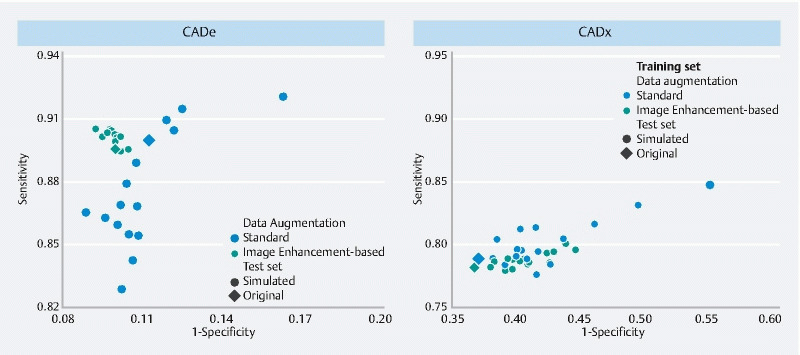
Performance variability of both computer-aided detection (CADe) and computer-aided diagnosis (CADx) using standard or image enhancement-based data augmentation. Each dot represents the performance of the respective CAD system on a test set comprising one specific enhancement setting.

All results are summarized in Table 1 and illustrated in Fig. 5 . Results on individual test sets are given in **Table 1 s** .

**Table 1:** Results of computer-aided detection and diagnosis systems trained with standard and image enhancement-based data augmentation.

AI application	Original test set	Simulated test sets		
		Median	Range (min–max)	*P* value
**CADe**				
Sensitivity, %				
Standard	90	87	9 (83–92)	< 0.001
Image enhancement	90	90	2 (89–91)	
Specificity, %				
Standard	89	89	7 (84–91)	0.003
Image enhancement	90	90	1 (90–91)	
**CADx**				
Sensitivity, %				
Standard	79	79	7 (78–85)	0.03
Image enhancement	78	79	2 (78–80)	
Specificity, %				
Standard	63	59	18 (45–63)	0.19
Image enhancement	63	60	8 (55–63)	

CADe, computer-aided detection; CADx, computer-aided diagnosis.

**Post hoc analysis** When the CADe system was trained with standard data augmentation using a different, more extreme enhancement setting (i. e. A1, A8, B1, or B8) and tested on all simulated test sets, performance variability increased even further. For instance, the CADe system trained solely on images with enhancement setting A8 displayed sensitivity scores between 66 % and 89 % (Δ 23 %). For CADx, the system trained on images with setting B1 reached specificity scores ranging between 31 % and 61 % (Δ 30 %). All post hoc results are displayed in **Table 2 s** , **Table 3 s** , and **Fig. 4 s** .

## Discussion

Current endoscopy systems offer a wide range of post-processing enhancement settings that endoscopist may use to adjust image characteristics such as color, texture, and contrast. We investigated whether these settings, while often subtle to the human eye, affect the performance of endoscopic AI systems. We tested this on two commonly used endoscopic AI applications, detection of early Barrett’s neoplasia and characterization of diminutive colorectal polyps, and found a remarkable impact of enhancement settings on AI performance.

When we trained and tested both CAD systems on their original datasets without changing enhancement settings of the original images, the systems performed as expected. However, when we evaluated the systems’ performances on test sets with different enhancement settings, performance varied substantially. For instance, the CADe system for detection of Barrett’s neoplasia showed sensitivity varying between 83 % and 92 %: a 9 % absolute difference in neoplasia detection. For the CADx system characterizing colorectal polyps, the variability in performance was even more profound, with specificity scores differing up to 18 %. When we trained the systems with the extremes of the enhancement settings, performance variability increased even more. For example, a CADe system trained only on images with setting B1 showed specificity between 55 % and 88 % depending on the enhancement setting of the test sets.

These findings are significant, as all major manufacturers of endoscopy systems offer a variety of company-specific enhancement modes. Often, these settings are preconfigured by the hospital’s technical services team. Most endoscopist do not adjust these settings further and many may not even be aware of them. As there is no clear default mode, these settings may differ widely between hospitals or even between individual endoscopy suites. Meanwhile, AI systems are typically neither trained nor optimized to perform consistently across the full range of these settings, which may contribute to the lack of generalizability observed when AI systems are deployed outside of the centers where they were initially developed 11 12 13 . For example, if an AI system trained on data with high-contrast settings is deployed in a clinic where low-contrast settings are standard, it may fail to detect subtle lesions due to the lower visual contrast, potentially leading to missed diagnoses. Conversely, other enhancement settings might amplify the AI system’s sensitivity, causing it to often flag non-neoplastic mucosa as suspicious. This could lead to a high false-positive rate, creating “alert fatigue” in clinicians.

Fortunately, there may be an effective solution to this issue. As the main problem is the lack of heterogeneity of enhancement settings in the training data, an intuitive solution is to introduce the complete spectrum of enhancement settings into the training data. Enhancement settings are based on post-processing transformations. Therefore, this does not require collection of additional real-world clinical data. Rather, the solution may be found with image enhancement-based data augmentation. Data augmentation is a standard step in training AI algorithms in endoscopy. It generally involves making slight modifications to the original images in the training set – such as rotations, color adjustments, or scaling – to artificially expand the dataset and introduce a wider variety of training examples. Image enhancement-based data augmentation involves restricting these augmentations to endoscopy-specific settings, in this case, the variability induced by enhancement settings. Using a proprietary software tool, we duplicated all images in the CAD systems’ training sets to create new images comprising all different enhancement settings. This approach proved to be successful, as both the CADe and CADx systems displayed significantly more stable performance: CADe sensitivity and specificity differences were limited to 2 % and 1 %, while CADx differences were 2 % and 8 %, respectively.

We recognize that this study has some limitations. First, this study is limited by its ex vivo design using simulated still images. Images were converted to different enhancement settings after initial acquisition and storage, using a proprietary software tool that exactly replicates these settings. Although a prospective collection of matched images with different enhancement settings would be ideal, it is impractical, if not impossible, to capture matched pairs across all enhancement settings for each image. Additionally, varying capture devices and compression techniques could introduce further variability, potentially distorting results. In contrast, our ex vivo approach allowed for a standardized, paired analysis across 16 enhancement settings. Moreover, as shown in **Fig. 2 s** , the converted images were nearly indistinguishable from the original data, supporting the validity of this approach. Second, despite the fact that the evaluated datasets were aimed at different endoscopic applications, the datasets originated from the same manufacturer and endoscope series. Other endoscopy systems were not investigated. Yet it is conceivable that AI systems on these platforms will suffer from similar issues. Third, the current proposed method for image enhancement-based data augmentation remains a quick fix and may not be sustainable long term. Ideally, a more generic method would be developed that is applicable to all current post-processing settings and future settings yet to be developed. Fourth, this study is specifically aimed at post-processing image enhancement. There may be other sources of domain shift that can form a similar threat to model generalizability, such as other virtual chromoendoscopy techniques, differing generations of endoscopes, and quality of the endoscopic imaging procedure.

In conclusion, this study highlights the impact of different enhancement settings on performance variability of endoscopic AI systems. This should be taken into account during future development and implementation of AI in endoscopy. Collecting more diverse data during the development phase may offer the most direct solution, but specific data augmentation techniques to simulate enhancement setting variability or generative AI solutions could be pragmatic alternatives and deserve further investigation.

## Supplementary Material

Supplementary material Zusatzmaterial
