# Supplementary material for: Impact of standard enhancement settings of endoscopy systems on performance of endoscopic artificial intelligence systems
Source: Endoscopy. 2025 Feb 28;57(6):602–10. doi: 10.1055/a-2530-1845 (PMC12119143; doi:10.1055/a-2530-1845)
Supplement: Supplementary file 1 — Supplementary material [file 24533supmat_10-1055-a-2530-1845.pdf]

Standard enhancement settings used on endoscopy systems significantly impair performance of artificial intelligence systems in endoscopy.

M.R. Jong, C.H.J. Kusters, Q.N.E. van Bokhorst, J.B. Jukema, R.A.H. van Eijck van Heslinga, K.N. Fockens, B.B.S.L. Houwen, T.J.M. Jaspers, T.G.W. Boers, M. van der Vlugt, E. Dekker, F. van der Sommen, P.H.N. de With, A.J. de Groof, J.J. Bergman on behalf of the BONS-AI Consortium.

TABLE OF CONTENT

- Members and collaborators of the BONSAI-consortium
- Algorithm development
- Supplementary Tables
- Supplementary Figures

Endoscopy | DOI 10.1055/a-2530-1845 | © The Author(s).

BONS-AI Consortium

The authors wish to thank the members and collaborators of the BONS-AI Consortium, as listed below in alphabetical order:

Alaa Alkhalaf, Isala Hospital, Zwolle, the Netherlands;

Lorenza Alvarez Herrero, St. Antonius Hospital, Utrecht, the Netherlands;

Francisco Baldaque-Silva, Karolinska University Hospital, Stockholm, Sweden;

Maximilien Barret, Cochin Hospital, Paris, France;

Jacques J Bergman, Amsterdam UMC, Amsterdam, the Netherlands;

Torsten Beyna, Evangelisches Krankenhaus Düsseldorf, Düsseldorf, Germany;

Tim G Boers, TU Eindhoven, Eindhoven, the Netherlands;

Lucas C Duits, Amsterdam UMC, Amsterdam, the Netherlands;

Rixta A H, Amsterdam UMC, Amsterdam, the Netherlands;

Peter Elbe, Karolinska University Hospital & Karolinska Institute, Stockholm, Sweden;

Kiki N Fockens, Amsterdam UMC, Amsterdam, the Netherlands;

Albert J de Groof, Amsterdam UMC, Amsterdam, the Netherlands;

Martin H Houben, HagaZiekenhuis, The Hague, the Netherlands;

Martijn R Jong, Amsterdam UMC, Amsterdam, the Netherlands;

Tim J M Jaspers, TU Eindhoven, Eindhoven, the Netherlands;

Jelmer B Jukema, Amsterdam UMC, Amsterdam the Netherlands;

Carolus H. J. Kusters, TU Eindhoven, Eindhoven, the Netherlands;

Rosalie C Mallant-Hent, Flevoziekenhuis, Almere, the Netherlands;

Guiomar Moral Villarejo, Nottingham University Hospitals NHS Trust, Nottingham, United Kingdom

Wouter Nagengast, UMC Groningen, Groningen, the Netherlands;

Jacobo Ortiz Fernández-Sordo, Nottingham University Hospitals NHS Trust, Nottingham, United Kingdom

Oliver Pech, St. John of God Hospital, Regensburg, Germany;

Roos E Pouw, Amsterdam UMC, Amsterdam, the Netherlands;

Joost A van der Putten, TU Eindhoven, Eindhoven, the Netherlands;

Krish Ragunath, Royal Perth Hospital, Perth, Australia;

Pieter Scholten, Onze Lieve Vrouwe Gasthuis, Amsterdam, the Netherlands;

Stefan Seewald, Klinik Hirslanden, Zurich, Switzerland;

Fons van der Sommen, TU Eindhoven, Eindhoven, the Netherlands;

Jessie Westerhof, UMC Groningen, Groningen, the Netherlands;

Bas L Weusten, UMC Utrecht & St. Antonius Hospital, Utrecht, the Netherlands;

Peter H de With, TU Eindhoven, Eindhoven, the Netherlands;

Endoscopy | DOI 10.1055/a-2530-1845 | © The Author(s).

Algorithm development

All CAD models (CADe and CADx, based on A1, A8, B1, B8 or all combined) were trained three times with a separate patient split (i.e. seeds). Seeds were the same among the different CAD systems. All systems were based on a ResNet-50 encoder with ImageNet initialized weights. Each model underwent training with a batch size of 32 samples across 50 epochs using the Adam optimizer and the Binary Cross-Entropy loss function. During training, positive and negative samples were randomly sampled to ensure an average 50:50 representation in each iteration, while for CADx the loss of negative samples was additionally weighted 4x higher than positive samples to counter the class imbalance. The initial learning rate was 1e-4 which was reduced by a factor 10 when the validation loss did not decrease for 10 epochs. Input images were downsampled to a resolution of 256x256 pixels.

Two different data augmentation regimens were applied during training to virtually increase the set size: standard and image enhancement-based data augmentation. Both included several basic transformations: horizontal and vertical flipping, rotation by $\theta \in \{0°, 90°, 180°, 270°\}$, Gaussian blurring, random affine, followed by random artificial Gaussian noise corruption. For ‘standard’ data augmentation, we also included contrast/saturation/brightness enhancements, gray-scale conversion and sharpness transforms. For ‘image enhancement-based’ data augmentation, we included images on the outer ends of the spectrum of available enhancement settings (i.e. A1, A8, B1 and B8).

During internal validation, operating thresholds were selected with criteria of sensitivity ≥ 90% and sensitivity ≥ 80% for CADe and CADx, respectively.

Endoscopy | DOI 10.1055/a-2530-1845 | © The Author(s).

Table 1s Results of CAD systems trained using standard or image enhancement-based data augmentation per training and test dataset.

Application	Data Augmentation	Enhancement Setting Test Set	Performance Metric	Score
CADe	Image Enhancement-based<br>Standard	A1	Sensitivity	90% (87 - 92)<br>87% (84 - 89)
CADe	Image Enhancement-based<br>Standard	A1	Specificity	90% (87 - 92)<br>89% (86 - 91)
CADe	Image Enhancement-based<br>Standard	A2	Sensitivity	90% (87 - 92)<br>88% (85 - 90)
CADe	Image Enhancement-based<br>Standard	A2	Specificity	90% (87 - 92)<br>90% (87 - 92)
CADe	Image Enhancement-based<br>Standard	A3	Sensitivity	90% (87 - 92)<br>89% (86 - 91)
CADe	Image Enhancement-based<br>Standard	A3	Specificity	90% (87 - 92)<br>89% (86 - 91)
CADe	Image Enhancement-based<br>Standard	A4	Sensitivity	90% (87 - 92)<br>90% (87 - 92)
CADe	Image Enhancement-based<br>Standard	A4	Specificity	90% (87 - 92)<br>88% (85 - 90)
CADe	Image Enhancement-based<br>Standard	A5	Sensitivity	90% (87 - 92)<br>90% (87 - 92)
CADe	Image Enhancement-based<br>Standard	A5	Specificity	90% (87 - 92)<br>89% (86 - 91)
CADe	Image Enhancement-based<br>Standard	A6	Sensitivity	90% (87 - 92)<br>91% (89 - 93)
CADe	Image Enhancement-based<br>Standard	A6	Specificity	90% (87 - 92)<br>88% (85 - 90)
CADe	Image Enhancement-based<br>Standard	A7	Sensitivity	89% (86 - 91)<br>91% (89 - 93)
CADe	Image Enhancement-based<br>Standard	A7	Specificity	90% (87 - 92)<br>87% (84 - 90)
CADe	Image Enhancement-based<br>Standard	A8	Sensitivity	90% (87 - 92)<br>92% (90 - 94)
CADe	Image Enhancement-based<br>Standard	A8	Specificity	90% (87 - 92)<br>84% (81 - 87)
CADe	Image Enhancement-based<br>Standard	B1	Sensitivity	90% (87 - 92)<br>83% (80 - 86)
CADe	Image Enhancement-based<br>Standard	B1	Specificity	90% (87 - 92)<br>90% (87 - 92)
CADe	Image Enhancement-based<br>Standard	B2	Sensitivity	90% (87 - 92)<br>84% (81 - 87)
CADe	Image Enhancement-based<br>Standard	B2	Specificity	90% (87 - 92)<br>89% (86 - 91)
CADe	Image Enhancement-based<br>Standard	B3	Sensitivity	90% (87 - 92)<br>85% (82 - 88)
CADe	Image Enhancement-based<br>Standard	B3	Specificity	90% (87 - 92)<br>89% (86 - 91)

Endoscopy | DOI 10.1055/a-2530-1845 | © The Author(s).

CADe	Image Enhancement-based<br>Standard	B4	Sensitivity	90% (87 - 92)<br>85% (82 - 88)
CADe	Image Enhancement-based<br>Standard	B4	Specificity	90% (87 - 92)<br>89% (86 - 91)
CADe	Image Enhancement-based<br>Standard	B5	Sensitivity	90% (87 - 92)<br>86% (83 - 88)
CADe	Image Enhancement-based<br>Standard	B5	Specificity	90% (87 - 92)<br>90% (87 - 92)
CADe	Image Enhancement-based<br>Standard	B6	Sensitivity	91% (89 - 93)<br>87% (84 - 89)
CADe	Image Enhancement-based<br>Standard	B6	Specificity	90% (87 - 92)<br>90% (87 - 92)
CADe	Image Enhancement-based<br>Standard	B7	Sensitivity	91% (89 - 93)<br>86% (83 - 88)
CADe	Image Enhancement-based<br>Standard	B7	Specificity	91% (88 - 93)<br>90% (87 - 92)
CADe	Image Enhancement-based<br>Standard	B8	Sensitivity	90% (87 - 92)<br>87% (84 - 89)
CADe	Image Enhancement-based<br>Standard	B8	Specificity	91% (88 - 93)<br>91% (88 - 93)
CADx	Image Enhancement-based<br>Standard	A1	Sensitivity	80% (76 - 83)<br>79% (75 - 82)
CADx	Image Enhancement-based<br>Standard	A1	Specificity	56% (47 - 65)<br>60% (51 - 69)
CADx	Image Enhancement-based<br>Standard	A2	Sensitivity	79% (75 - 82)<br>80% (76 - 83)
CADx	Image Enhancement-based<br>Standard	A2	Specificity	57% (48 - 66)<br>60% (51 - 69)
CADx	Image Enhancement-based<br>Standard	A3	Sensitivity	79% (75 - 82)<br>80% (76 - 83)
CADx	Image Enhancement-based<br>Standard	A3	Specificity	58% (49 - 67)<br>62% (53 - 71)
CADx	Image Enhancement-based<br>Standard	A4	Sensitivity	79% (75 - 82)<br>81% (78 - 84)
CADx	Image Enhancement-based<br>Standard	A4	Specificity	59% (50 - 68)<br>60% (51 - 69)
CADx	Image Enhancement-based<br>Standard	A5	Sensitivity	79% (75 - 82)<br>81% (78 - 84)
CADx	Image Enhancement-based<br>Standard	A5	Specificity	60% (51 - 69)<br>58% (49 - 67)
CADx	Image Enhancement-based<br>Standard	A6	Sensitivity	78% (74 - 81)<br>82% (79 - 85)
CADx	Image Enhancement-based<br>Standard	A6	Specificity	62% (53 - 71)<br>54% (45 - 63)
CADx	Image Enhancement-based<br>Standard	A7	Sensitivity	79% (75 - 82)<br>83% (80 - 86)
CADx	Image Enhancement-based<br>Standard	A7	Specificity	62% (53 - 71)<br>50% (41 - 59)
CADx	Image Enhancement-based<br>Standard	A8	Sensitivity	79% (75 - 82)<br>85% (82 - 88)

Endoscopy | DOI 10.1055/a-2530-1845 | © The Author(s).

CADx	Image Enhancement-based<br>Standard	A8	Specificity	61% (52 - 70)<br>45% (36 - 54)
CADx	Image Enhancement-based<br>Standard	B1	Sensitivity	80% (76 - 83)<br>78% (74 - 81)
CADx	Image Enhancement-based<br>Standard	B1	Specificity	55% (46 - 64)<br>58% (49 - 67)
CADx	Image Enhancement-based<br>Standard	B2	Sensitivity	78% (74 - 81)<br>78% (74 - 81)
CADx	Image Enhancement-based<br>Standard	B2	Specificity	57% (48 - 66)<br>61% (52 - 70)
CADx	Image Enhancement-based<br>Standard	B3	Sensitivity	78% (74 - 81)<br>79% (75 - 82)
CADx	Image Enhancement-based<br>Standard	B3	Specificity	59% (50 - 68)<br>59% (50 - 68)
CADx	Image Enhancement-based<br>Standard	B4	Sensitivity	78% (74 - 81)<br>79% (75 - 82)
CADx	Image Enhancement-based<br>Standard	B4	Specificity	60% (51 - 69)<br>60% (51 - 69)
CADx	Image Enhancement-based<br>Standard	B5	Sensitivity	78% (74 - 81)<br>79% (75 - 82)
CADx	Image Enhancement-based<br>Standard	B5	Specificity	60% (51 - 69)<br>62% (53 - 71)
CADx	Image Enhancement-based<br>Standard	B6	Sensitivity	79% (75 - 82)<br>79% (75 - 82)
CADx	Image Enhancement-based<br>Standard	B6	Specificity	60% (51 - 69)<br>58% (49 - 67)
CADx	Image Enhancement-based<br>Standard	B7	Sensitivity	78% (74 - 81)<br>78% (74 - 81)
CADx	Image Enhancement-based<br>Standard	B7	Specificity	61% (52 - 70)<br>57% (48 - 66)
CADx	Image Enhancement-based<br>Standard	B8	Sensitivity	79% (75 - 82)<br>80% (76 - 83)
CADx	Image Enhancement-based<br>Standard	B8	Specificity	59% (50 - 68)<br>56% (47 - 65)

Endoscopy | DOI 10.1055/a-2530-1845 | © The Author(s).

Table 2s Results of post hoc analysis of CAD systems trained on more extreme enhancement settings.

Application	Enhancement setting Training Set	Metric	Simulated Test Sets	
			Median	Range (min-max)
CADe	A1	Sensitivity	89%	9% (86-95)
		Specificity	91%	24% (69-94)
	A8	Sensitivity	78%	23% (66-89)
		Specificity	96%	8% (90-99)
	B1	Sensitivity	91%	5% (88-94)
		Specificity	78%	33% (55-88)
	B8	Sensitivity	88%	10% (83-93)
		Specificity	93%	16% (80-96)
CADx	A1	Sensitivity	79%	10% (76-86)
		Specificity	61%	25% (40-65)
	A8	Sensitivity	80%	6% (76-82)
		Specificity	55%	20% (44-64)
	B1	Sensitivity	78%	11% (74-85)
		Specificity	58%	30% (31-61)
	B8	Sensitivity	79%	4% (78-82)
		Specificity	56%	12% (47-59)

Endoscopy | DOI 10.1055/a-2530-1845 | © The Author(s).

Table 3s Results of post hoc analysis of CAD system trained on more extreme enhancement settings per training and test dataset.

Application	Enhancement Setting Training Set	Enhancement Setting Test Set	Performance Metric	Score
CADe	A1<br>A8<br>B1<br>B8	A1	Sensitivity	89% (86 - 91)<br>76% (73 - 79)<br>90% (87 - 92)<br>87% (84 - 89)
CADe	A1<br>A8<br>B1<br>B8	A1	Specificity	90% (87 - 92)<br>97% (95 - 98)<br>83% (80 - 86)<br>95% (93 - 96)
CADe	A1<br>A8<br>B1<br>B8	A2	Sensitivity	90% (87 - 92)<br>79% (76 - 82)<br>91% (89 - 93)<br>88% (85 - 90)
CADe	A1<br>A8<br>B1<br>B8	A2	Specificity	90% (87 - 92)<br>96% (94 - 97)<br>82% (79 - 85)<br>95% (93 - 96)
CADe	A1<br>A8<br>B1<br>B8	A3	Sensitivity	90% (87 - 92)<br>82% (79 - 85)<br>91% (89 - 93)<br>89% (86 - 91)
CADe	A1<br>A8<br>B1<br>B8	A3	Specificity	88% (85 - 90)<br>95% (93 - 96)<br>79% (75 - 82)<br>93% (91 - 95)
CADe	A1<br>A8<br>B1<br>B8	A4	Sensitivity	92% (90 - 94)<br>85% (82 - 88)<br>91% (89 - 93)<br>91% (89 - 93)
CADe	A1<br>A8<br>B1<br>B8	A4	Specificity	87% (84 - 90)<br>94% (92 - 96)<br>75% (71 - 78)<br>91% (88 - 93)
CADe	A1<br>A8<br>B1<br>B8	A5	Sensitivity	92% (90 - 94)<br>84% (81 - 87)<br>91% (89 - 93)<br>90% (87 - 92)
CADe	A1<br>A8<br>B1<br>B8	A5	Specificity	85% (82 - 88)<br>95% (93 - 96)<br>74% (70 - 77)<br>91% (88 - 93)
CADe	A1<br>A8<br>B1<br>B8	A6	Sensitivity	93% (91 - 95)<br>86% (83 - 88)<br>92% (90 - 94)<br>91% (89 - 93)
CADe	A1<br>A8<br>B1<br>B8	A6	Specificity	82% (79 - 85)<br>93% (91 - 95)<br>69% (65 - 73)<br>88% (85 - 90)
CADe	A1<br>A8	A7	Sensitivity	94% (92 - 96)<br>87% (84 - 89)

Endoscopy | DOI 10.1055/a-2530-1845 | © The Author(s).

	B1			93% (91 - 95)
	B8			92% (90 - 94)
CADe	A1	A7	Specificity	78% (74 - 81)
	A8			92% (89 - 94)
	B1			64% (60 - 68)
	B8			86% (83 - 89)
CADe	A1	A8	Sensitivity	95% (93 - 96)
	A8			89% (86 - 91)
	B1			94% (92 - 96)
	B8			93% (91 - 95)
CADe	A1	A8	Specificity	69% (65 - 73)
	A8			90% (87 - 92)
	B1			55% (51 - 59)
	B8			80% (77 - 83)
CADe	A1	B1	Sensitivity	86% (83 - 88)
	A8			66% (62 - 69)
	B1			88% (85 - 90)
	B8			83% (80 - 86)
CADe	A1	B1	Specificity	91% (88 - 93)
	A8			99% (98 - 100)
	B1			88% (85 - 90)
	B8			96% (94 - 97)
CADe	A1	B2	Sensitivity	86% (83 - 88)
	A8			69% (65 - 72)
	B1			89% (86 - 91)
	B8			84% (81 - 87)
CADe	A1	B2	Specificity	92% (89 - 94)
	A8			98% (97 - 99)
	B1			87% (84 - 90)
	B8			95% (93 - 96)
CADe	A1	B3	Sensitivity	87% (84 - 89)
	A8			73% (70 - 76)
	B1			90% (87 - 92)
	B8			85% (82 - 88)
CADe	A1	B3	Specificity	91% (88 - 93)
	A8			97% (95 - 98)
	B1			86% (83 - 89)
	B8			95% (93 - 96)
CADe	A1	B4	Sensitivity	87% (84 - 89)
	A8			75% (72 - 78)
	B1			90% (87 - 92)
	B8			86% (83 - 88)
CADe	A1	B4	Specificity	93% (91 - 95)
	A8			97% (95 - 98)
	B1			85% (82 - 88)
	B8			95% (93 - 96)
CADe	A1	B5	Sensitivity	88% (85 - 90)
	A8			78% (75 - 81)
	B1			91% (89 - 93)
	B8			87% (84 - 89)
CADe	A1	B5	Specificity	94% (92 - 96)
	A8			96% (94 - 97)
	B1			81% (78 - 84)
	B8			94% (92 - 96)
CADe	A1	B6	Sensitivity	89% (86 - 91)
	A8			78% (75 - 81)

Endoscopy | DOI 10.1055/a-2530-1845 | © The Author(s).

	B1			91% (89 - 93)
	B8			88% (85 - 90)
CADe	A1	B6	Specificity	93% (91 - 95)
	A8			96% (94 - 97)
	B1			77% (73 - 80)
	B8			93% (91 - 95)
CADe	A1	B7	Sensitivity	89% (86 - 91)
	A8			77% (74 - 80)
	B1			91% (89 - 93)
	B8			88% (85 - 90)
CADe	A1	B7	Specificity	94% (92 - 96)
	A8			96% (94 - 97)
	B1			76% (72 - 79)
	B8			92% (89 - 94)
CADe	A1	B8	Sensitivity	90% (87 - 92)
	A8			77% (74 - 80)
	B1			92% (90 - 94)
	B8			89% (86 - 91)
CADe	A1	B8	Specificity	92% (89 - 94)
	A8			96% (94 - 97)
	B1			70% (66 - 74)
	B8			91% (88 - 93)
CADx	A1	A1	Sensitivity	80% (76 - 83)
	A8			80% (76 - 83)
	B1			79% (75 - 82)
	B8			78% (74 - 81)
CADx	A1	A1	Specificity	61% (52 - 70)
	A8			54% (45 - 63)
	B1			60% (51 - 69)
	B8			56% (47 - 65)
CADx	A1	A2	Sensitivity	80% (76 - 83)
	A8			80% (76 - 83)
	B1			79% (75 - 82)
	B8			78% (74 - 81)
CADx	A1	A2	Specificity	62% (53 - 71)
	A8			55% (46 - 64)
	B1			57% (48 - 66)
	B8			59% (50 - 68)
CADx	A1	A3	Sensitivity	80% (76 - 83)
	A8			79% (75 - 82)
	B1			78% (74 - 81)
	B8			78% (74 - 81)
CADx	A1		Specificity	58% (49 - 67)
	A8			59% (50 - 68)
	B1	A3		57% (48 - 66)
	B8			59% (50 - 68)
CADx	A1	A4	Sensitivity	81% (78 - 84)
	A8			78% (74 - 81)
	B1			78% (74 - 81)
	B8			78% (74 - 81)
CADx	A1	A4	Specificity	56% (47 - 65)
	A8			62% (53 - 71)
	B1			55% (46 - 64)
	B8			57% (48 - 66)
CADx	A1	A5	Sensitivity	81% (78 - 84)
	A8			78% (74 - 81)

Endoscopy | DOI 10.1055/a-2530-1845 | © The Author(s).

	B1			80% (76 - 83)
	B8			78% (74 - 81)
CADx	A1	A5	Specificity	53% (44 - 62)
	A8			64% (55 - 72)
	B1			51% (42 - 60)
	B8			56% (47 - 65)
CADx	A1	A6	Sensitivity	83% (80 - 86)
	A8			77% (73 - 80)
	B1			81% (78 - 84)
	B8			79% (75 - 82)
CADx	A1	A6	Specificity	50% (41 - 59)
	A8			64% (55 - 72)
	B1			45% (36 - 54)
	B8			55% (46 - 64)
CADx	A1	A7	Sensitivity	83% (80 - 86)
	A8			76% (72 - 79)
	B1			83% (80 - 86)
	B8			79% (75 - 82)
CADx	A1	A7	Specificity	46% (37 - 55)
	A8			64% (55 - 72)
	B1			40% (31 - 49)
	B8			53% (44 - 62)
CADx	A1	A8	Sensitivity	86% (83 - 89)
	A8			77% (73 - 80)
	B1			85% (82 - 88)
	B8			82% (79 - 85)
CADx	A1	A8	Specificity	40% (31 - 49)
	A8			64% (55 - 72)
	B1			31% (23 - 40)
	B8			47% (38 - 56)
CADx	A1	B1	Sensitivity	78% (74 - 81)
	A8			81% (78 - 84)
	B1			78% (74 - 81)
	B8			78% (74 - 81)
CADx	A1	B1	Specificity	62% (53 - 71)
	A8			44% (35 - 53)
	B1			58% (49 - 67)
	B8			52% (43 - 61)
CADx	A1	B2	Sensitivity	77% (73 - 80)
	A8			82% (79 - 85)
	B1			78% (74 - 81)
	B8			79% (75 - 82)
CADx	A1	B2	Specificity	65% (56 - 73)
	A8			45% (36 - 54)
	B1			58% (49 - 67)
	B8			54% (45 - 63)
CADx	A1	B3	Sensitivity	77% (73 - 80)
	A8			82% (79 - 85)
	B1			78% (74 - 81)
	B8			79% (75 - 82)
CADx	A1	B3	Specificity	63% (54 - 72)
	A8			46% (37 - 55)
	B1			58% (49 - 67)
	B8			54% (45 - 63)
CADx	A1	B4	Sensitivity	76% (72 - 79)
	A8			82% (79 - 85)

Endoscopy | DOI 10.1055/a-2530-1845 | © The Author(s).

	B1			77% (73 - 80)
	B8			79% (75 - 82)
CADx	A1	B4	Specificity	64% (55 - 72)
	A8			49% (40 - 58)
	B1			59% (50 - 68)
	B8			56% (47 - 65)
CADx	A1	B5	Sensitivity	76% (72 - 79)
	A8			80% (76 - 83)
	B1			76% (72 - 79)
	B8			79% (75 - 82)
CADx	A1	B5	Specificity	63% (54 - 72)
	A8			51% (42 - 60)
	B1			61% (52 - 70)
	B8			58% (49 - 67)
CADx	A1	B6	Sensitivity	77% (73 - 80)
	A8			81% (78 - 84)
	B1			75% (71 - 78)
	B8			79% (75 - 82)
CADx	A1	B6	Specificity	62% (53 - 71)
	A8			53% (44 - 62)
	B1			58% (49 - 67)
	B8			58% (49 - 67)
CADx	A1	B7	Sensitivity	76% (72 - 79)
	A8			80% (76 - 83)
	B1			74% (70 - 77)
	B8			79% (75 - 82)
CADx	A1	B7	Specificity	62% (53 - 71)
	A8			56% (47 - 65)
	B1			60% (51 - 69)
	B8			57% (48 - 66)
CADx	A1	B8	Sensitivity	79% (75 - 82)
	A8			78% (74 - 81)
	B1			75% (71 - 78)
	B8			79% (75 - 82)
CADx	A1	B8	Specificity	58% (49 - 67)
	A8			56% (47 - 65)
	B1			55% (46 - 64)
	B8			58% (49 - 67)

Endoscopy | DOI 10.1055/a-2530-1845 | © The Author(s).

Fig. 1s Example of the full range of enhancement settings of the EXERA III system (Olympus, Tokyo). Both type A and type B enhance fine mucosal patterns.

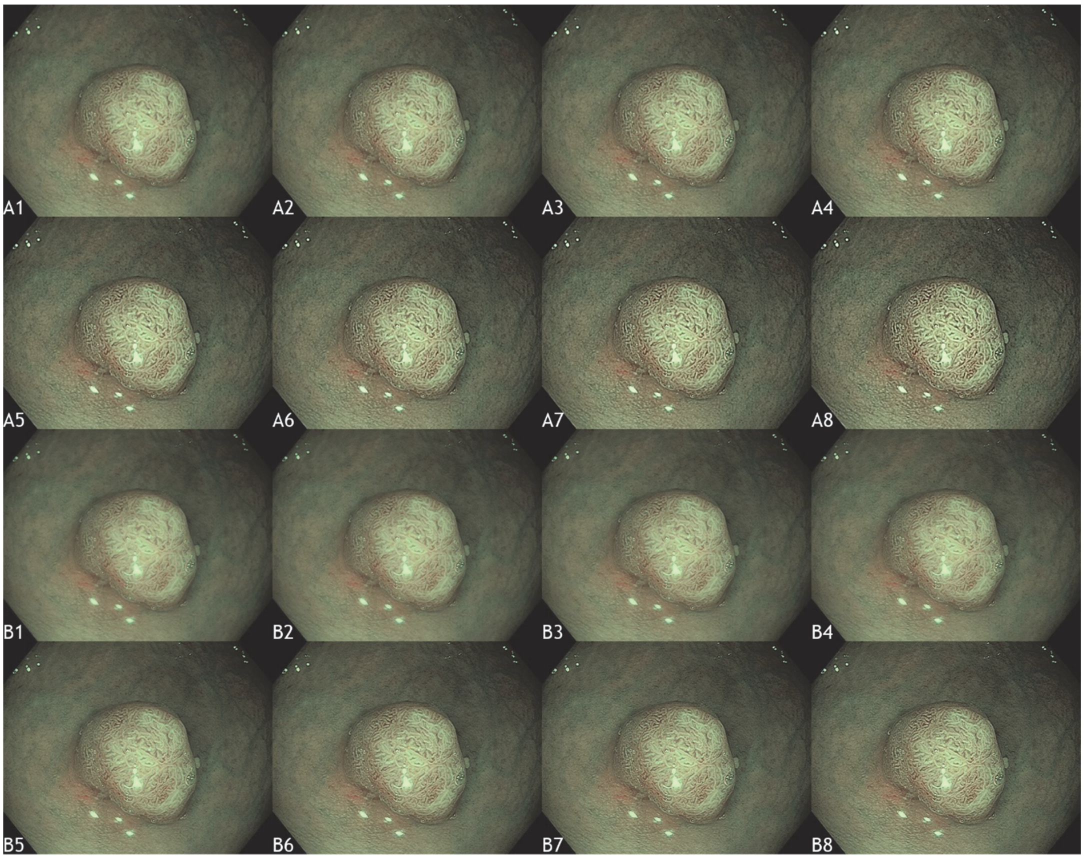

Endoscopy | DOI 10.1055/a-2530-1845 | © The Author(s).

Fig. 2s Validation data of the conversion software. Original images were captured with setting A1 and A5. The software tool was then used to convert the A5 image artificially to A1. Differences in RGB pixel values between original images and generated images are given on the right.

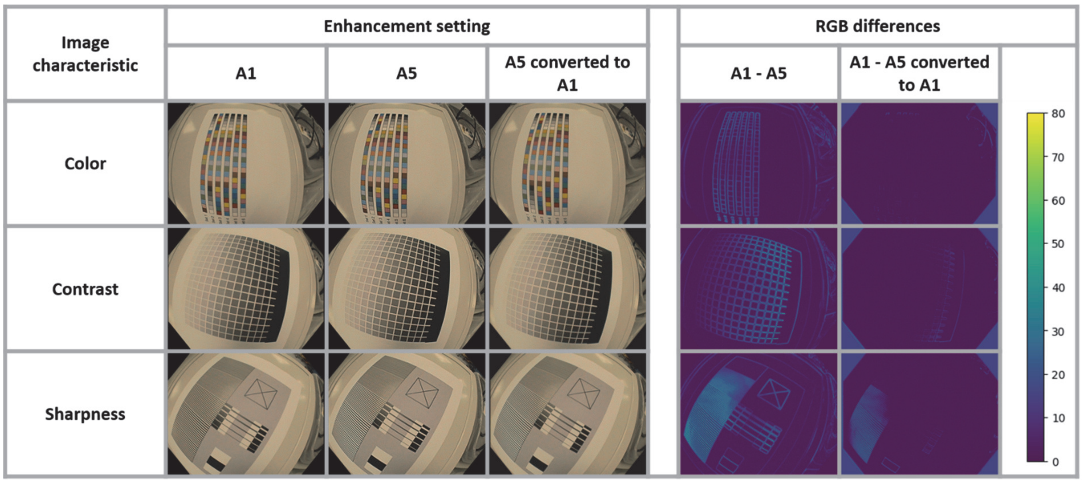

Fig. 3s Examples of data augmentation techniques: 1) geometric transformations such as vertical and horizontal flipping, random rotation and shearing; 2) filtering, e.g. sharpness and Gaussian blur; 3) color transformations, e.g. contrast, hue and brightness adjustments; and 4) image enhancement-based data augmentations, e.g. similar images with different enhancement settings.

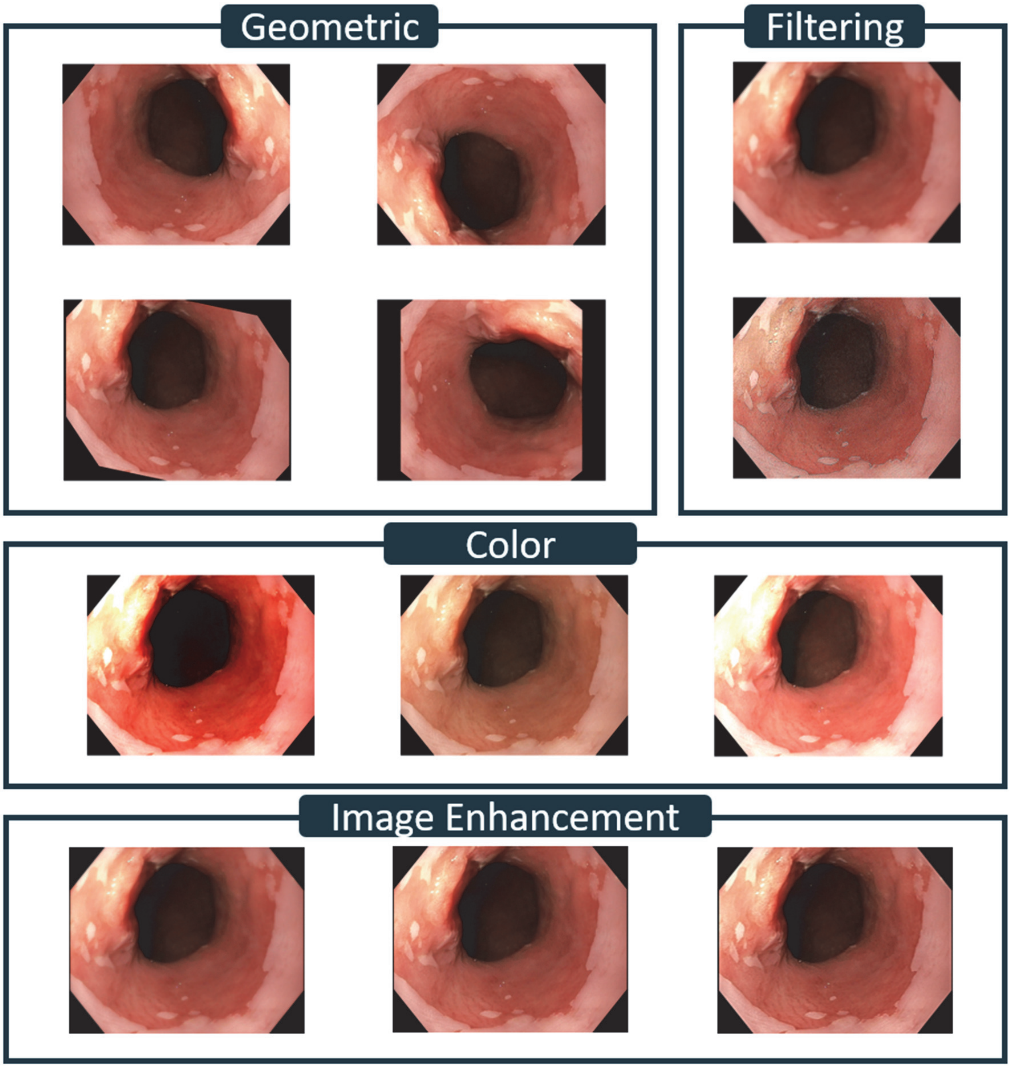

Endoscopy | DOI 10.1055/a-2530-1845 | © The Author(s).

Fig. 4s Performance variability of both CADe and CADx, trained on enhancement setting A1, A8, B1, or B8. Each dot represents the performance of the respective CAD system on a test set comprising one specific enhancement setting.

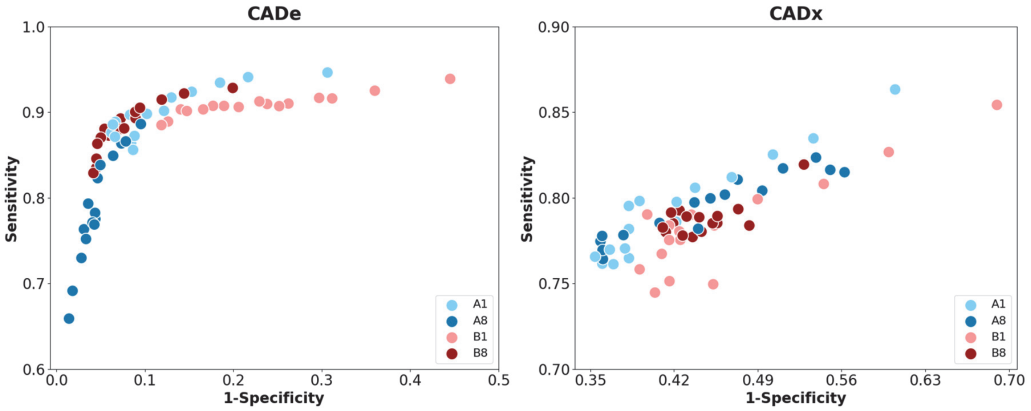

Endoscopy | DOI 10.1055/a-2530-1845 | © The Author(s).
